# The involvement of actin, calcium channels and exocytosis proteins in somato-dendritic oxytocin and vasopressin release

**DOI:** 10.3389/fphys.2012.00261

**Published:** 2012-07-12

**Authors:** Vicky Tobin, Gareth Leng, Mike Ludwig

**Affiliations:** Centre for Integrative Physiology, University of EdinburghEdinburgh, UK

**Keywords:** exocytosis, hypothalamus, pituitary, vasopressin, oxytocin, neuropeptides

## Abstract

Hypothalamic magnocellular neurons release vasopressin and oxytocin not only from their axon terminals into the blood, but also from their somata and dendrites into the extracellular space of the brain, and this can be regulated independently. Differential release of neurotransmitters from different compartments of a single neuron requires subtle regulatory mechanisms. Somato-dendritic, but not axon terminal release can be modulated by changes in intracellular calcium concentration [(Ca^2+^)] by release of calcium from intracellular stores, resulting in priming of dendritic pools for activity-dependent release. This review focuses on our current understanding of the mechanisms of priming and the roles of actin remodeling, voltage-operated calcium channels (VOCCs) and SNARE proteins in the regulation somato-dendritic and axon terminal peptide release.

## Introduction

Neurons have classically been considered to propagate information in one direction; synaptic inputs onto the dendrites or soma initiate action potentials which, after conduction to the axon terminal, transmit information to the postsynaptic neuron via a neurochemical signal that is confined to the pre- and post-synaptic area. However, neurochemicals can ‘spillover’ to have extra-synaptic actions, and in some cases can be released from dendrites. Peptides in particular have actions unlikely to be confined to synapses: they are packaged in large dense-cored vesicles (LDCVs), containing much more cargo than conventional synaptic vesicles; they have much higher affinities for their receptors than conventional neurotransmitters, half-lives much longer than conventional neurotransmitters, and in general are not conspicuously located at synapses but are present throughout the cell (Leng and Ludwig, 2008). More than 100 peptides are expressed and secreted by different neuronal populations throughout the brain, and many neuropeptides have profound effects on specific behaviors. These considerations imply that neuropeptides have organizational and activational roles that make them more akin to hormones than to classical neurotransmitters (Ludwig and Leng, 2006).

**Figure 1:**
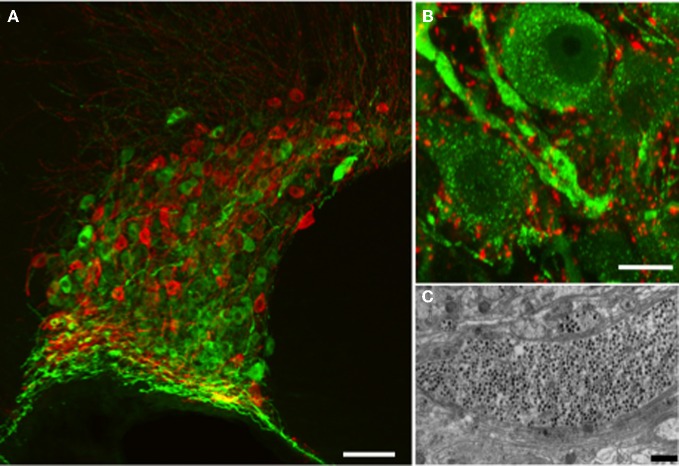
**Vasopressin and oxytocin are synthesized by a few thousand large (magnocellular) neurons (vasopressin cells are immunostained with fluorescent green and oxytocin cells with fluorescent red) whose cell bodies are located mainly in the supraoptic (A) and paraventricular (not shown) nuclei of the hypothalamus. (B)** The vasopressin immunostaining is punctate and represents individual or aggregates of large dense-cored vesicles. In the dendrite thickenings the vesicles are particularly abundant. Strong punctuate staining of VAMP2 (red labeling) was seen around the vasopressin and oxytocin (not shown) somata and dendrites, however there was no co-localization with the peptide suggesting labeling of pre-synaptic terminals. **(C)** Large dense-core vesicles in an electron microscopic section of a dendrite appear as dark, round, membrane-bound organelles (black dots). Scale bars show **(A)** 100, **(B)** 10, and **(C)** 1μm respectively.

Among the best-established sites of dendritic release are the supraoptic (SON) and paraventricular nuclei (PVN) of the hypothalamus, where magnocellular neurons synthesize vasopressin and oxytocin. These peptides are packaged into LDCVs that are abundant in the soma and dendrites as well as in swellings and nerve endings in the neurohypophysis (Figure 1; Leng and Ludwig, 2008). These neurons are aggregated into relatively homogeneous nuclei, and the SON is particularly homogeneous, containing only magnocellular vasopressin and oxytocin neurons, so studies of dendritic release from the SON can be accomplished in the absence of contamination by axonal release of peptide.

Somato-dendritic release was first unequivocally confirmed in this system using tannic acid fixation and electron microscopy; this allowed the visualization of omega fusion profiles in the dendritic plasma membrane and the dense-cores from exocytosed vesicles in the extracellular space (Morris and Pow, 1991). These studies also showed that magnocellular neurons lack active zones – the specialized region of the presynaptic terminal at which exocytosis typically occurs (Pow and Morris, 1989; Morris and Pow, 1991). Indeed they showed that exocytosis could occur from any part of the neuron with the probability of release from any compartment determined simply by the number of vesicles that were close to the plasma membrane.

The blood-brain barrier is impermeable to oxytocin and vasopressin, and simultaneous measurement of peptide release within the blood and the brain has demonstrated that release from these compartments can be independently controlled (Ludwig and Leng, 2006). For example, in lactating rats, suckling evokes intermittent pulsatile secretion of oxytocin into the blood to mediate milk let-down, and this is the result of synchronous bursting discharge of the oxytocin neurons. However, suckling stimulates dendritic oxytocin release *before* peripheral secretion occurs, and this is essential for co-ordinating the bursting activity (Moos et al., 1989; Rossoni et al., 2008). By contrast, systemic osmotic stimulation activates vasopressin neurons and increases secretion of vasopressin from the pituitary, but dendritic vasopressin release is delayed, occurring after plasma concentrations of vasopressin have returned to baseline (Ludwig et al., 1994).

Over the last decade, *in vivo* and *in vitro* studies have revealed many aspects of the control of dendritic vasopressin and oxytocin release (Landgraf, 1995; Ludwig, 1998; Ludwig and Pittman, 2003; Landgraf and Neumann, 2004). Here we focus on the roles of actin remodeling, voltage operated calcium channels (VOCCs) and SNARE proteins in the regulation of somato-dendritic and axon terminal release.

## Autoregulation and priming

**Figure 2:**
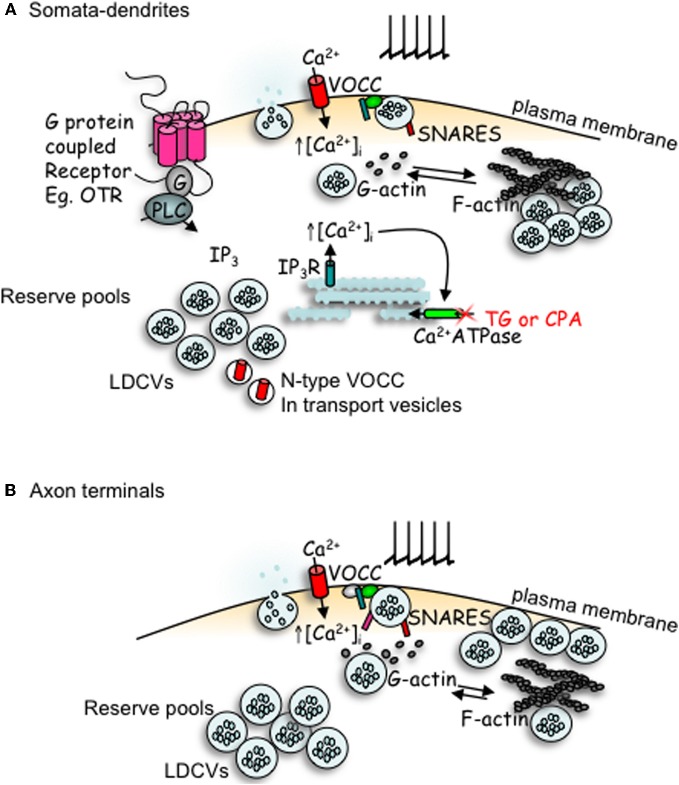
**Comparison of peptides release from somata-dendrites (A) and axon terminals (B) of magnocellular neurons.** Depolarization induced calcium entry via voltage-operated calcium channels (VOCCs) stimulates peptide release from large dense-cored vesicles (LDCVs). In the somata-dendrites this requires the depolymerization of F-actin to G-actin. The stimulation of G-protein coupled receptors, such as the oxytocin receptor (OTR), stimulates the mobilization of calcium from intracellular stores and an increase in both the number of LDCVs and N-type channels at the plasma membrane which primes release for subsequent activity-dependent release. In contrast, release from axon terminal appears more simple; LDCV movement utilizes actin depolymerization, but release does not depend upon it. Although some members of the SNARE family are detectable by immunocytochemistry in both compartments, there appears to be a lack of VAMP, SNAP-25 and synaptotagmin-1 in the somata-dendrites, with their function perhaps being replaced by other SNARE proteins.

Exocytosis of oxytocin and vasopressin from the neurohypophysis results from calcium entry via voltage-gated channels following depolarization of the terminals by invading action potentials (Leng et al., 1999) (Figure 2). By contrast, some chemical signals, notably oxytocin itself, can elicit dendritic release without increasing the electrical activity of the neurons. In particular, activation of G-protein coupled receptors on the dendrites can elevate intracellular [Ca^2+^] enough to trigger exocytosis of LDCVs from the soma and dendrites (Figure 2). Oxytocin neurons express oxytocin receptors (Freund-Mercier et al., 1994), and activation of these receptors mobilises calcium from thapsigargin-sensitive intracellular stores, producing a rise in intracellular [Ca^2+^] that can trigger dendritic oxytocin release (Lambert et al., 1994). Thus, once triggered, dendritic oxytocin release can be self-sustaining and hence long-lasting (Ludwig and Leng, 2006). This self-sustaining nature of oxytocin release and its physiological role has been demonstrated in parturient rats. During parturition, oxytocin is released from the SON and this drives the pulsatile release of oxytocin into the periphery to cause uterine contractions and thus regulate pup delivery. Infusion of an oxytocin receptor antagonist into the SON during parturition significantly reduced SON oxytocin release, and delayed further pup delivery (Neumann et al., 1996).

As vasopressin neurons similarly express receptors for vasopressin, part of the function of dendritic release involves auto-regulation of neuronal activity, either by acting directly (Gouzenes et al., 1998), or indirectly, by regulating afferent inputs (Kombian et al., 1997, 2002; Curras-Collazo et al., 2003). For oxytocin neurons, this presynaptic action is partly mediated by oxytocin-induced production of endocannabinoids (Hirasawa et al., 2004), acting at CB1 receptors on presynaptic glutamatergic terminals. These effects act on different spatial and temporal scales, and one important consequence is the emergence of intense, synchronous bursting activity, the key phenomenon that underpins the milk-ejection reflex (Rossoni et al., 2008). For vasopressin cells, the autoregulatory effects are different, but are also complex, because vasopressin is inhibitory to active vasopressin cells but excitatory to inactive cells (Gouzenes et al., 1998). Thus, vasopressin release tends to reduce the heterogeneity of firing rates amongst vasopressin cells, and this may be an important load-sharing mechanism during sustained secretory demand, such as dehydration (Leng et al., 2008b).

How much dendritic release occurs in response to electrical activity depends on the extent to which the vesicle pools in the dendrites are available for release. In magnocellular neurons, increases in intracellular [Ca^2+^] induced by agents such as thapsigargin or cyclopiazonic acid, which block calcium re-uptake into intracellular calcium stores and hence result in a large, transient increase in intracellular [Ca^2+^], result in the preparation (“priming”) of dendritic vesicle pools for subsequent activity-dependent release (Ludwig et al., 2002, 2005) (Figure 2). This priming is not a consequence of elevation of intracellular [Ca^2+^] *per se*, as priming is detected well after the increase in intracellular [Ca^2+^] has returned to baseline levels (Lambert et al., 1994), and as depolarization-induced increases in intracellular [Ca^2+^] do not result in priming. Priming involves *preparing* a system for some anticipated trigger that will come at some uncertain time in the future; it involves making the secretory pool of the target cell available for rapid release in response to that future trigger. In particular, oxytocin binding to oxytocin neurons has been shown to prime dendritic oxytocin release (Ludwig et al., 2002).

Analogous priming occurs in some endocrine cells. In the anterior pituitary of oestrogen-primed female rats, luteinising hormone releasing hormone (LHRH) is capable of “self-priming” gonadotrophs, causing a potentiation of luteinising hormone release in response to successive challenges with LHRH. This priming is delayed and long-lasting, and involves translocation of LDCVs to docking sites at the plasma membrane (Thomas and Clarke, 1997; Leng et al., 2008a). Priming in magnocellular neurons similarly involves a recruitment of LDCVs from a reserve pool into a readily-releasable pool (Tobin et al., 2004), but also involves a recruitment of VOCCs (Tobin et al., 2011). Priming does not appear to require either *de novo* transcription or translation (Tobin and Ludwig, 2007a).

## F-actin

Because peptide release from magnocellular neurons is not restricted to any particular part of the plasma membrane (Morris and Pow, 1991), regulation depends on controlling the access of vesicles to the plasma membrane, and in endocrine cells this control is exerted by cytoskeletal elements (Morgan, 1995; Park and Loh, 2008). Many secretory cells possess a network of cortical polymerized actin (filamentous or F-actin) in the subplasmalemal space. Cortical F-actin is often described as a barrier, as it is thought to anchor the LDCVs and regulate their availability for docking at the plasma membrane (Goddette and Frieden, 1986; Vitale et al., 1995; Ehre et al., 2005). Consistent with this idea, F-actin undergoes fast, transient and reversible depolymerization during exocytosis from many cells (Cheek and Burgoyne, 1986; Nakata and Hirokawa, 1992; Trifaro et al., 2000) and areas of exocytosis have been shown to be void of F-actin (Goddette and Frieden, 1986; Nakata and Hirokawa, 1992). In general, release of neurotransmitters from axon terminals is increased after F-actin depolymerization (Morales et al., 2000; Sankaranarayanan et al., 2003). Similarly, in chromaffin cells, depolymerization of F-actin increases the translocation of vesicles to the plasma membrane (Vitale et al., 1995) and polymerization of actin inhibits exocytosis (Zhang et al., 1995). However, depolymerization of F-actin *inhibits* release from PC12 cells (Matter et al., 1989), HIT insulin-secreting cells (Li et al., 1994) and mast cells (Pendleton and Koffer, 2001). Thus, F-actin can also facilitate vesicle fusion for release, depending on the cell type or time course of measured response.

As well as a network throughout the cytoplasm, the cell bodies of magnocellular neurons possess a network of F-actin beneath the plasma membrane (Tobin and Ludwig, 2007b). Actin depolymerization can stimulate peptide release from both the dendritic and axonal compartments, consistent with the idea of cortical actin acting as a barrier for LDCVs to access release sites. Concomitant actin polymerization or depolymerization does not affect secretion from the axon terminals, but release from the dendrites is inhibited by actin polymerization and potentiated by actin depolymerization. Thus, depolarization-evoked release from the dendrites, unlike that from the axon terminals, *requires* actin depolymerization. It has previously been suggested that a cortical F-actin network might separate vesicles into a small readily releasable pool and a larger reserve pool (Trifaro et al., 2000).

The role of actin in regulating the availability of LDCVs for dendritic release is highlighted by the observation that actin depolymerization potentiates depolarization-evoked dendritic release, yet blocks thapsigargin-induced priming. In hippocampal neurons, F-actin polymerization potentiates thapsigargin-induced increases in intracellular [Ca^2+^] (Wang et al., 2002), so it seems unlikely that the block of priming is because of an effect on calcium mobilization.

F-actin might facilitate release either by providing “tracks” that permit the docking of vesicles at appropriate membrane sites, or by spatially constraining components of the exocytotic machinery. This suggests that activation of release involves a reorganization of F-actin which allows the vesicles access to the exocytotic sites and provides the structural support necessary for exocytosis. In the magnocellular system, it appears that F-actin remodeling regulates the availability of mature and readily-releasable vesicles in different parts of the cell, and thus may be involved in the differential control of release from different parts of the cell.

## Voltage operated calcium channels

Like axon terminal release, dendritic release of oxytocin and vasopressin depends on the entry of calcium into the cell (Neumann et al., 1993; Shibuya et al., 1998; de Kock et al., 2003) via VOCCs (Fisher and Bourque, 1996). Whereas terminal secretion is very sensitive to the frequency of action potentials, dendritic release is normally less tightly coupled to action potential events (Leng and Ludwig, 2008), but depolarization-induced dendritic release can be primed by a prior mobilization of intracellular calcium (Ludwig et al., 2002, 2005). This mechanism is absent from the axon terminals of magnocellular neurones, which lack thapsigargin-sensitive calcium stores. In some systems, neuronal synaptic release of neurotransmitters can be potentiated by a recruitment of VOCCs to the active zone, increasing calcium entry upon depolarization. Thus, one target for priming may be a change in the number and/or activity of VOCCs or an increased proximity of channels to docked vesicles (Becherer et al., 2003).

Magnocellular neurons express several types of VOCC (Foehring and Armstrong, 1996; Joux et al., 2001), but one subtype in particular, the N-type channels, appears to be particularly important for dendritic release. Although the current carried by N-type channels is comparatively minor in magnocellular somata compared to the other VOCC types or indeed the whole-cell calcium current (Joux et al., 2001; Tobin et al., 2011), release of oxytocin from SONs is most sensitive to blockade of N-type channels. This suggests that a change in the expression and/or activity of VOCCs might underlie priming, and indeed, thapsigargin treatment significantly increases the calcium current carried by N-type channels as a proportion of the whole-cell calcium current (Tobin et al., 2011).

As mentioned, priming does not require *de novo* transcription or translation, arguing against new channels or channel constituents being made. Therefore, the potential mechanisms are either that existing N-type channels at the plasma membrane carry more charge per channel, or that more N-type channels are inserted into the membrane. The latter is more likely, as there is no change in the voltage-dependent activity of the N-type channels after priming, and suggests that there is a “reserve pool” of channels (Tobin et al., 2011).

We observed a strong perinuclear immunocytochemical signal for N-type channels in both oxytocin and vasopressin somata. Differentiated neuroblastoma, neuronal and endocrine cell types all contain an intracellular pool of recruitable N-type channels which can be translocated to the plasma membrane (Passafaro et al., 1994, 1998; Sher et al., 1998). This suggests the presence of a channel reserve pool via which a neuron can increase the number of cell surface VOCCs, for example during synaptogenesis or, as in this case, to increase secretory responsiveness. In neuroendocrine bag cell neurons of *Aplysia californica*, actin-dependent translocation of VOCCs from the central region of a growth cone into the plasma membrane of the growth cone is an important part of the process of transformation into mature neurosecretory endings (Knox et al., 1992; Zhang et al., 2008). Recruitment of N-type channels on peptide-containing LDCVs seems unlikely, as there is not a strong co-localization of the N-type channel and either oxytocin or vasopressin (Tobin et al., 2011). Thus, the priming signal stimulates the translocation of both peptide-containing LDCVs and N-type channels in parallel.

The particular involvement of one type of VOCC with a secretory response has previously been demonstrated with the involvement of L-type channels in somato-dendritic vasopressin release by pituitary adenylate cyclase activating polypeptide (PACAP) (Shibuya et al., 1998), R-type channels with the axon terminal release of oxytocin (Wang et al., 1999) and P/Q-type channels with vasopressin secretion (Wang et al., 1997). The requirement for somato-dendritic release of calcium entry through mainly L- and N-type channels has been shown for other transmitters, including dynorphin (Simmons et al., 1995), dopamine (Kim et al., 2009; Mendez et al., 2011) and serotonin (De-Miguel and Trueta, 2005).

As previously reviewed (Catterall, 2000; Felix, 2005) VOCC activity can be acutely modulated by events such as phosphorylation/de-phosphorylation or by interaction (via G-proteins) with other membrane receptors. These rapid modulatory events occur, and revert, within a time-scale of a few milliseconds to minutes. By contrast, gonadotrophs also show a steroid-dependent modulation of VOCCs which occurs over a much longer time-scale (24–36 h). Treating gonadotrophs with estradiol produces a time-dependent change in secretory responsiveness which mimics the pre-ovulatory luteinizing hormone surge. This treatment also stimulates a parallel change in the calcium current (Heyward and Clarke, 1995) that depends on the synthesis of new VOCCs and their insertion into the plasma membrane, and this change is a prerequisite for gonadotrophs to display the self-priming response to LHRH. Here, we suggest that a stimulus that produces an increased secretory responsiveness with an intermediate time scale (30–90 min) may cause magnocellular neurons to recruit N-type channels to the plasma membrane, allowing them to respond to a subsequent depolarization with a larger secretory response.

## SNARE proteins

The stimulated release of both LDCVs and synaptic vesicles involves the N-ethylmaleimide sensitive fusion protein attachment protein receptor (SNARE) complex, which allows the vesicle membrane to fuse with the plasma membrane. The roles in dendritic release of the protein members which comprise this complex have been reviewed recently (Ovsepian and Dolly, 2011). The basic configuration of this complex comprises a vesicle associated membrane protein-2 (VAMP2), syntaxin-1 and soluble N-ethylmaleimide attachment protein-25 (SNAP25) (or other members of the VAMP, syntaxin or SNAP families). Other proteins regulate the activity of these core proteins (e.g., munc18 regulating syntaxin-1), or act as calcium sensors (e.g., synaptotagmin-1). Some of these proteins, including VAMP-2, are targeted to exocytosis sites by being inserted into the coat of the vesicle, others, including RIM, bassoon and piccolo, are targeted to the pre-synaptic area and assembled into specialized zones of release by the cytomatrix proteins (tom Dieck et al., 1998; Fenster et al., 2000).

Despite their ability to release LDCVs, the magnocellular neuron dendrites showed a surprising lack of some of these core proteins. We (Tobin et al., 2012) and others (Deleuze et al., 2005) did not detect any immunocytochemical signal for VAMP-2 despite abundant signal surrounding these cells on pre-synaptic contacts (Figure 1). In fact, we did not detect co-localization of any VAMP family member with either peptide, as would be expected for a protein inserted into the vesicle coat. We also failed to detect SNAP-25, but did detect syntaxin-1 and munc-18. Although we detected all of these in the axon terminals, the resolution did not allow us to determine if the terminal VAMP-2 was associated with LDCVs. An earlier study using electron microscopy also failed to demonstrate an association between LDCVs and VAMP-2 in the terminals (Zhang et al., 2000).

Although they express VAMP-2 and SNAP-25, gonadotrophs lack an immunocytochemically-detectable signal for syntaxin-1 (Thomas et al., 1998). Knock-out of syntaxin 1A was not lethal in mice, and although synaptic plasticity was impaired, basic synaptic release appeared normal (Fujiwara et al., 2006). Although knock-out of either SNAP-25 or VAMP-2 in mice was lethal after birth, *in vitro* recordings from embryonic brains showed compromised but not ablated evoked synaptic release, as well as reduced spontaneous release. Significantly in all three cases, there was no compensatory expression of other SNARE proteins (Schoch et al., 2001; Washbourne et al., 2002). Perhaps there are more members or isoforms of the existing members to be identified, but for the time being somato-dendritic peptide release from these magnocellular neurons and anterior pituitary gonadotrophs appear to occur in the absence of the full complement of exocytotic machinery considered to be mandatory.

### Conflict of interest statement

The authors declare that the research was conducted in the absence of any commercial or financial relationships that could be construed as a potential conflict of interest.
